# The *FgHOG1* Pathway Regulates Hyphal Growth, Stress Responses, and Plant Infection in *Fusarium graminearum*

**DOI:** 10.1371/journal.pone.0049495

**Published:** 2012-11-14

**Authors:** Dawei Zheng, Shijie Zhang, Xiaoying Zhou, Chenfang Wang, Ping Xiang, Qian Zheng, Jin-Rong Xu

**Affiliations:** 1 NWAFU-PU Joint Research Center, College of Plant Protection, Northwest A&F University, Yangling, Shaanxi, China; 2 Department of Botany and Plant Pathology, Purdue University, West Lafayette, Indiana, United States of America; Seoul National University, Republic of Korea

## Abstract

Fusarium head blight (FHB) caused by *Fusarium graminearum* is a destructive disease of wheat and barley worldwide. In a previous study of systematic characterization of protein kinase genes in *F. graminearum*, mutants of three putative components of the osmoregulation MAP kinase pathway were found to have distinct colony morphology and hyphal growth defects on PDA plates. Because the osmoregulation pathway is not known to regulate aerial hyphal growth and branching, in this study we further characterized the functions of the FgHog1 pathway in growth, pathogenesis, and development. The *Fghog1*, *Fgpbs2*, and *Fgssk2* mutants were all reduced in growth rate, aerial hyphal growth, and hyphal branching angle. These mutants were not only hypersensitive to osmotic stress but also had increased sensitivity to oxidative, cytoplasm membrane, and cell wall stresses. The activation of FgHog1 was blocked in the *Fgpbs2* and *Fgssk2* mutants, indicating the sequential activation of FgSsk2-FgPbs2-FgHog1 cascade. Interestingly, the FgHog1 MAPK pathway mutants appeared to be sensitive to certain compounds present in PDA. They were female sterile but retained male fertility. We also used the metabolomics profiling approach to identify compatible solutes that were accumulated in the wild type but not in the *Fghog1* deletion mutant. Overall, our results indicate that the FgSsk2-FgPbs2-FgHog1 MAPK cascade is important for regulating hyphal growth, branching, plant infection, and hyperosmotic and general stress responses in *F. graminearum*.

## Introduction

*Fusarium graminearum* is a causal agent of Fusarium head blight (FHB) or scab of wheat and barley [1], [2]. Under favorable conditions, this pathogen can cause severe yield losses and contaminate infested grains with harmful mycotoxins such as deoxynivalenol (DON) and zearalenones. Like many other plant diseases caused by *Fusarium* species, FHB is difficult to control due to the lack of type I resistance genes and the complexity of resistance in identified germplasms [3], [4]. In addition, cost effective control of FHB by fungicide application remains to be developed.

In fungi and other eukaryotic organisms, a family of serine/threonine protein kinases known as mitogen-activated protein (MAP) kinases is involved in the regulation of different growth and developmental processes in response to a variety of extracellular signals. The MAP kinase (MAPK) is activated by MAP kinase kinase (MEK), which is phosphorylated by MEK kinase (MEKK). Sequential activation of the MEKK-MEK-MAPK cascade results in the expression of specific sets of genes in response to environmental stimuli. The budding yeast *Saccharomyces cerevisiae* has five MAP kinase pathways that are involved in pheromone response, filamentation, sporulation, osmoregulation, and cell wall integrity. The high osmolarity glycerol (HOG) response pathway is required for growth under hyperosmotic conditions [5], [6]. The yeast Hog1 MAPK is activated by the Pbs2 MEKK, which in turn is activated by two overlapping MEKKs, Ssk2, and Ssk22. A two component histidine kinase system functions upstream from Ssk2/Ssk22 for response to hyperosmotic stress [7]. Pbs2 also can be activated by Ste11 via a putative membrane protein Sho1 [8] and a transmembrane mucin [9].

The osmoregulation MAPK pathway is well conserved in fungi. Among all the fungi that have been sequenced, only the intracellular parasitic microsporidium *Encephalitozoon cuniculi* lacks Hog1 ortholog and other MAPK genes [10]. Like p38 and other stress-activated MAP kinases, Hog1 and its orthologs have the TGY dual phosphorylation site. Although the Hog1 MAPK pathway is mainly involved in osmoregulation in *S. cerevisiae*, its orthologs often have additional functions in various biological functions in filamentous fungi [11]. In a number of fungi, this MAPK cascade is involved in responses to oxidative stresses and sensitivities to dicarboximide and phenylpyrrole fungicides [12], [13], [14]. It also has been shown to be important for responses to cell wall stresses and SDS in certain fungi, including *Aspergillus fumigatus* and *Botrytis cinerea* [15], [16]. In plant pathogenic fungi such as *Magnaporthe oryzae*, this MAPK pathway is dispensable for plant infection [17]. However, it is essential for pathogenesis in *Mycosphaerella graminicola*, *B. cinerea*, *Bipolaris oryzae*, and other fungi [12], [13], [18]. In *F. graminearum*, the *FgOS-2* pathway mutants were reported to be defective in growth under hyperosmotic conditions and resistant to the dicarboximide and phenylpyrrole fungicides [19]. They were reduced in DON production and *TRI5* gene expression in rice grain cultures. However, the function of this MAPK pathway in other developmental and plant infection processes was not characterized [19].

In a previous study, we systematically characterized the predicted protein kinase genes in *F. graminearum* [20], including all the genes related to three MAPK cascades that are well conserved in filamentous fungi. To our surprise, the mutants defective in the osmoregulation pathway were found to have distinct colony morphology and hyphal growth defects. In this study, we characterized the functions of the FgHog1 pathway in growth under different conditions, sexual and asexual reproduction, DON production, and plant infection and confirmed the functional relationship among *FgHOG1*, *FgPBS2*, and *FgSSK2*. In addition to reduced growth rate, the *Fghog1*, *Fgpbs2*, and *Fgssk2* mutants were defective in aerial hyphal growth and hyphal branching. These mutants lost female fertility and failed to accumulate compatible solutes in response to NaCl treatment. They also had increased sensitivity to oxidative and cytoplasm membrane stresses and were defective in spreading through the rachis of infected wheat heads. Therefore, the FgHog1 pathway is involved in hyphal growth, branching, plant infection, and stress responses in *F. graminearum*.

## Results

### The *FgSSK2*, *FgPBS2*, and *FgHOG1* genes are important for hyphal growth in *F. graminearum*

The FGSG_00408, FGSG_08691, and FGSG_09612 genes are orthologous to the MEKK, MEK, and MAPK genes of the HOG pathway in the budding yeast [5] and named as *FgSSK2*, *FgPBS2*, and *FgHOG1*, respectively, in this study. The *Fgssk2*, *Fgpbs2*, and *Fghog1* mutants (Table 1) were generated by the split-marker approach described in the systematic characterization of protein kinase genes in *F. graminearum* [20]. At least two independent mutants were isolated for each gene and confirmed by Southern blot analysis. Although data were presented for only one of the mutants for each gene, all the mutant strains deleted of the same gene had the same phenotypes described below.

**Table 1:** Wild-type and mutant strains of *Fusarium graminearum* used in this study.

Strains	Brief description	Reference
PH-1	Wild-type	[54]
FK13	*Fgssk2* deletion mutant of PH-1	[20]
FK16	*Fgssk2* deletion mutant of PH-1	[20]
FK17	*Fgssk2* deletion mutant of PH-1	[20]
PS15	*Fgpbs2* deletion mutant of PH-1	[20]
PS1B1	*Fgpbs2* deletion mutant of PH-1	[20]
HG3	*Fghog1* deletion mutant of PH-1	[20]
HG6	*Fghog1* deletion mutant of PH-1	[20]
HG15	*Fghog1* deletion mutant of PH-1	[20]
FgMat2	*MAT1-2* deletion mutant of PH-1	This study
HGC1	*Fghog1/FgHOG1* complemented transformant	This study

**Figure 1:**
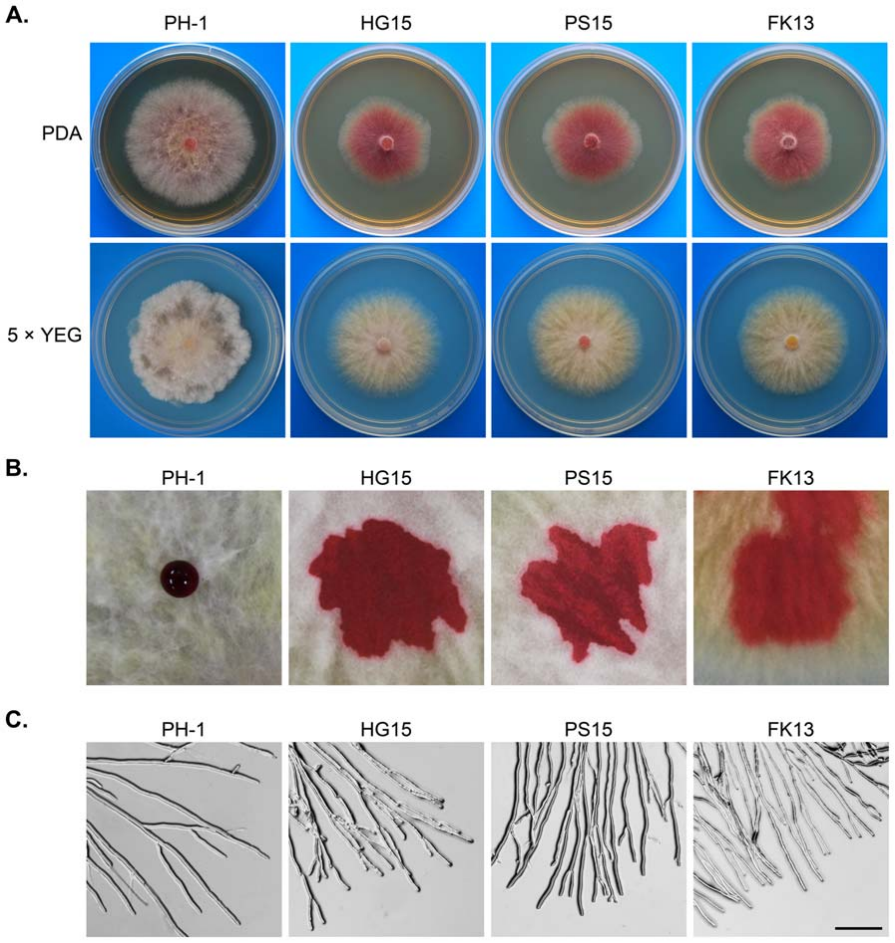
Growth defects of the *Fghog1*, *Fgpbs2*, and *Fgssk2* mutants. **A**. Colonies of the wild type (PH-1) and the *Fghog1* (HG15), *Fgpbs2* (PS15), and *Fgssk2* (FK13) mutants grown on PDA and 5xYEG agar plates for 3 days. **B**. Colony surface hydrophobicity tests with the same set of mutants. Photos were taken 15 min. after placing droplets of 50 µl red ink on the surface of the wild-type and mutant colonies. **C**. Hyphal tip growth and branching patterns of PH-1 and the same set of mutants on PDA plates. The branching angles were reduced in the extension zone of mutant colonies. Bar = 150 µm.

The *Fgssk2*, *Fgpbs2*, and *Fghog1* mutants were reduced in growth rate (Table 2) and had distinct colony morphology on PDA plates (Fig. 1A). To determine whether their growth defects are medium-dependent, we also assayed vegetative growth of the *Fgssk2*, *Fgpbs2*, and *Fghog1* mutants on CM and 5xYEG media. Although their growth rate also was reduced (Table 2), these mutants produced more aerial hyphae on CM or 5xYEG plates than on PDA plates. However, aerial hyphal growth still was reduced in comparison with that of the wild type (Fig. 1A). Furthermore, aerial hyphae produced by the mutants were less compact and had reduced surface hydrophobicity (Fig. 1B).

**Table 2:** Phenotypes of the *Fgssk2*, *Fgpbs2*, and *Fghog1* mutants in growth, conidiation, and plant infection.

Strain	Growth rate (mm/day)^a^			Conidiation (×10^5^/ml)	DON/Erg^b^	Disease index^c^
	PDA	CM	5xYEG			
PH-1	9.9±0.1^A^	8.9±0.3^A^	9.3±0.1^A^	9.90±3.00^A^	0.28	13.62
FK13	6.2±0.1^B^	7.4±0.1^B^	7.7±0.1^B^	5.30±1.50^C^	0.02	0.54
PS15	6.7±0.0^B^	7.6±0.1^B^	7.9±0.1^B^	4.70±1.80^C^	0.01	0.75
HG15	6.6±0.4^B^	7.7±0.1^B^	8.0±0.3^B^	7.70±2.40^B^	0.01	1.50
HGC1	10.1±0.5^A^	8.8±0.3^A^	9.2±0.1^A^	10.50±2.20^A^	0.33	N/A

a Growth rate and conidiation were measured after incubating for 3 and 5 days, respectively. Mean and standard deviation were calculated from three replicates. The same letter indicated there was no significant difference. Different letters were used to mark statistically significant differences (P = 0.05).

b DON/Ergosterol ratio was determined with 2-week-old rice grain cultures. Ergosterol was measured to quantify fungal biomass.

c N/A, not assayed.

Microscopic examination of hyphae grown on PDA revealed that the FgHog1 pathway mutants had a reduced branching angle. On the edge of PDA cultures, many hyphal branches of these mutants grew in parallel and in close proximity to each other at the extension zone (Fig. 1C). In contrast, most of the hyphal branches had the branching angle over 45 degrees in the wild-type strain PH-1 (Fig. 1C). The neighboring hyphal tips grew away from each other because of negative autotropism. These results indicate that the FgHog1 MAPK pathway plays a role in growth rate and hyphal branching in *F. graminearum*.

To determine whether nutritional conditions affected hyphal growth and colony morphologies in the *Fghog1* mutant, we supplemented PDA with all the components of 5xYEG to the same concentration of regular 5xYEG medium. On PDA+5xYEG plates, the *Fgssk2*, *Fgpbs2*, and *Fghog1* mutants had similar colony morphology defects as on regular PDA plates (Fig. S1).

### The FgHog1 pathway is important for regulating responses to hyperosmotic and cell membrane stresses

**Figure 2:**
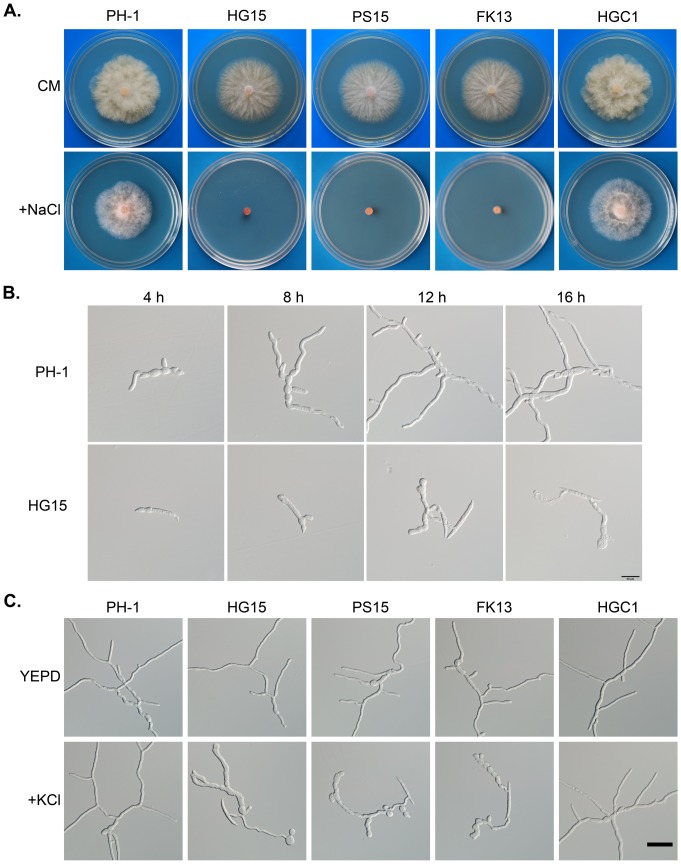
Defects of the *Fghog1*, *Fgpbs2*, and *Fgssk2* mutants in response to hyperosmotic stress. **A**. Colonies of PH-1, the *Fghog1* (HG15), *Fgpbs2* (PS15), and *Fgssk2* (FK13) mutants, and the *Fghog1*/*FgHOG1* complemented transformant (HGC1) on CM plates with or without 1 M NaCl. **B**. Conidium germination of PH-1 and the *Fghog1* mutant in CM with 0.7 M NaCl examined at 3 h, 12 h, and 18 h. Bar = 20 µm. **C**. Germlings of PH-1, HG15, PS15, FK13, and HGC1 incubated in CM+1 M KCl for 12 h. Bar = 40 µm.

On CM with 1 M NaCl, the *Fgssk2*, *Fgpbs2*, and *Fghog1* mutants had no obvious growth after incubation for 3 days (Fig. 2A). Conidium germination was delayed in the presence of 1 M NaCl (Fig. 2B). Whereas most of the wild-type conidia germinated by 4 h, no germination was observed in the *Fghog1* mutant. Furthermore, germ tube growth was severely stunted by NaCl treatment in the *Fghog1* mutant (Fig. 2B). In the presence of 1 M NaCl, germ tubes tended to be curvy and had irregular apical and subapical swelling in the mutants. Similar results were obtained when conidia of the *Fgssk2*, *Fgpbs2*, and *Fghog1* mutants were germinated in CM with 1 M KCl (Fig. 2C). These results indicate that the osmoregulation MAPK cascade is conserved in *F. graminearum* for regulating responses to hyperosmotic stress.

**Figure 3:**
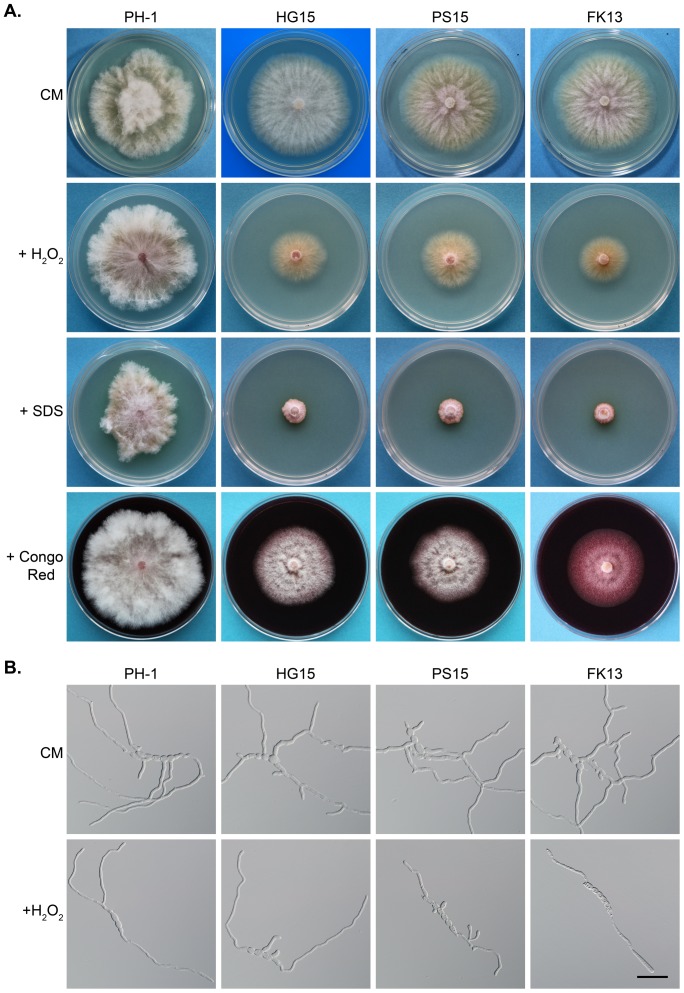
The FgHog1 MAPK pathway also is involved in responses to oxidative, cytoplasm membrane, and cell wall stresses. **A**. Colonies of PH-1 and the *Fghog1* (HG15), *Fgpbs2* (PS15), and *Fgssk2* (FK13) mutants on media with 0.05% H_2_O_2_, SDS, and Congo red. **B**. Germ tubes of the *Fghog1*, *Fgpbs2*, and *Fgssk2* mutants incubated in liquid YEPD with 0.005% H_2_O_2_. Bar = 40 µm.

Because this MAPK pathway is known to be involved in response to oxidative stress in several fungi [13], [21], we assayed the effects of H_2_O_2_ treatment on the *Fgssk2*, *Fgpbs2*, and *Fghog1* mutants. In the presence of 0.05% H_2_O_2_, all of these mutants had reduced growth rate (Fig. 3A). Microscopic examination revealed that H_2_O_2_ treatment also resulted in germ tube growth defects in the *Fgssk2*, *Fgpbs2*, and *Fghog1* mutants (Fig. 3B) but to a less content in comparison with NaCl treatment. We also assayed the effects of SDS and Congo red treatments that mimic cytoplasm membrane and cell wall stresses, respectively. In the presence of 0.05% SDS, colonial growth was stunted in the *Fgssk2*, *Fgpbs2*, and *Fghog1* mutants (Fig. 3A). However, unlikely 1 M NaCl cultures, aerial hyphal growth was still visible in SDS cultures (Fig. 3A). After incubated for 6 days, the colony diameter of SDS cultures could reach 1 cm in these mutants. Congo red treatment had no obvious effect on growth rate although aerial hyphae were reduced in the FgHog1 pathway mutants (Fig. 3A). These results suggest that the FgHog1 MAPK pathway also is involved in responses to oxidative and cytoplasm membrane stresses in *F. graminearum*.

### The *FgHOG1* pathway mutants were defective in sexual reproduction

**Figure 4:**
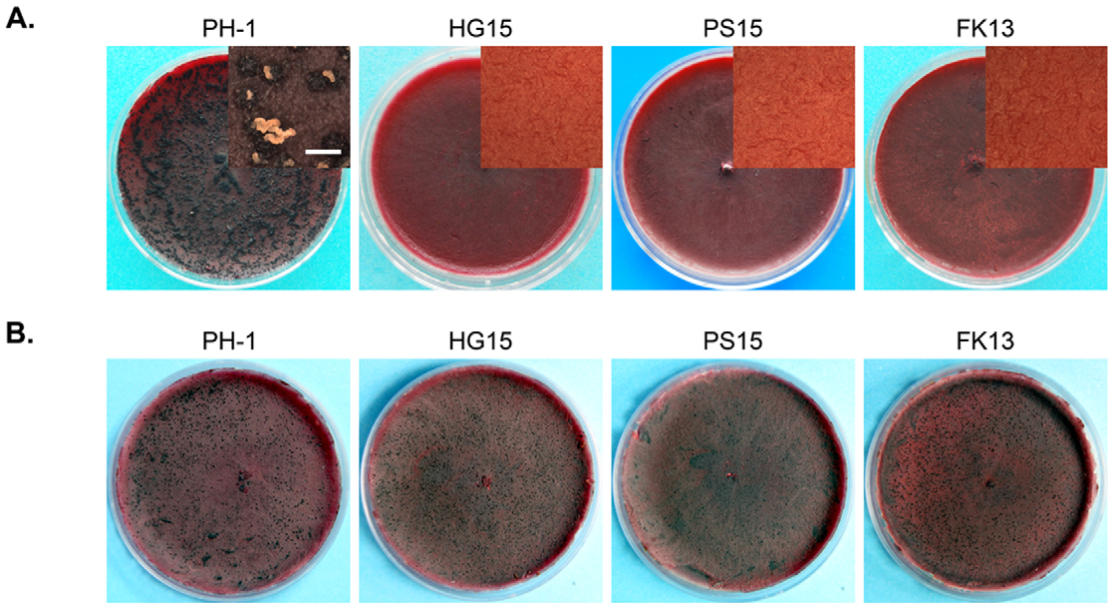
Defects of the *Fghog1*, *Fgpbs2*, and *Fgssk2* mutants in sexual reproduction. **A**. Self-crossing cultures of the wild type (PH-1) and the *Fghog1* (HG15), *Fgpbs2* (PS15), and *Fgssk2* (FK13) mutants. Fertile perithecia with cirrhi were only observed with the wild type. **B**. Carrot agar cultures of the *mat2* mutant fertilized with *Fghog1*, *Fgpbs2*, and *Fgssk2* mutants. All the mutants retained male fertility. The close-up views were taken under a dissecting microscope.

On self-mating carrot agar cultures, the *Fgssk2*, *Fgpbs2*, and *Fghog1* mutants were sterile. Whereas the wild type produced abundant perithecia with cirrhi two weeks after self-fertilization, perithecium formation was not observed in the mutant cultures incubated under the same conditions (Fig. 4A). Instead of sexual development, the FgHog1 pathway mutants appeared to be stimulated for asexual reproduction on fertilized carrot agar plates. Mutant cultures produced abundant conidia and sporodochia but lacked protoperithecium development.

To distinguish whether the FgHog1 pathway mutants were defective in male or female fertility, we conducted outcrossing assays with the *Fgssk2*, *Fgpbs2*, and *Fghog1* mutants. When they were used as the male to fertilize a *mat1-2* deletion strain, fertile perithecia were produced 7 days after fertilization (Fig. 4B), indicating that these mutants are normal in male fertility. Therefore, the FgHog1 MAPK pathway is essential for female fertility but dispensable for male fertility.

### The *FgHOG1* pathway is important for plant infection

**Figure 5:**
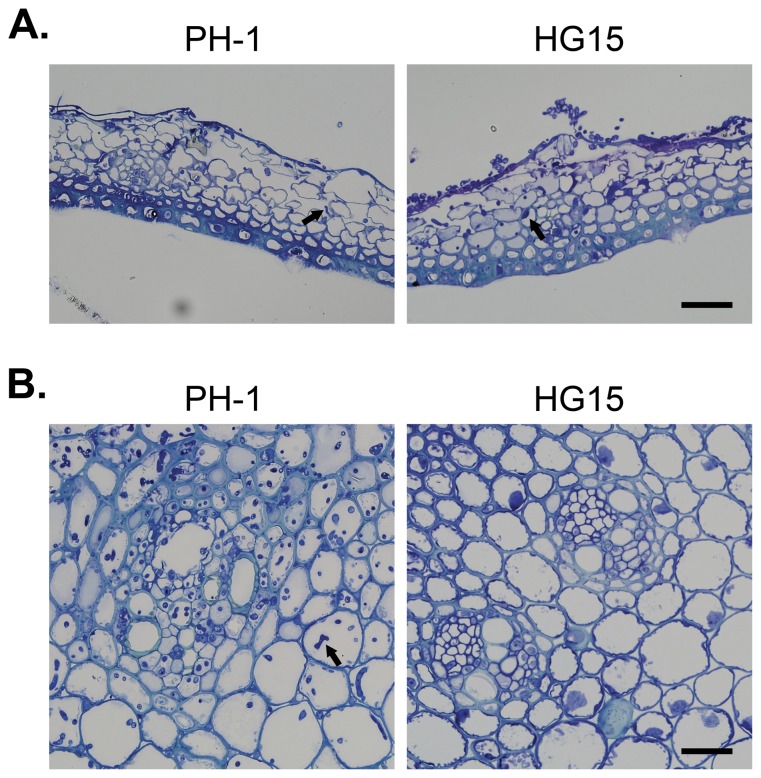
Flowering wheat heads were inoculated with the wild type (PH-1) and *Fghog1* mutant (HG15). **A**. Colonization of glume tissues by PH-1 and HG15 was examined 48 hpi. **B**. The rachises directly beneath the inoculated spikeletes were examined 120 hpi. Hyphae growth (marked with arrows) was abundant in plant tissues inoculated with PH-1 and but not in samples inoculated with *Fghog1* mutant. Bar = 40 µm.

**Figure 6:**
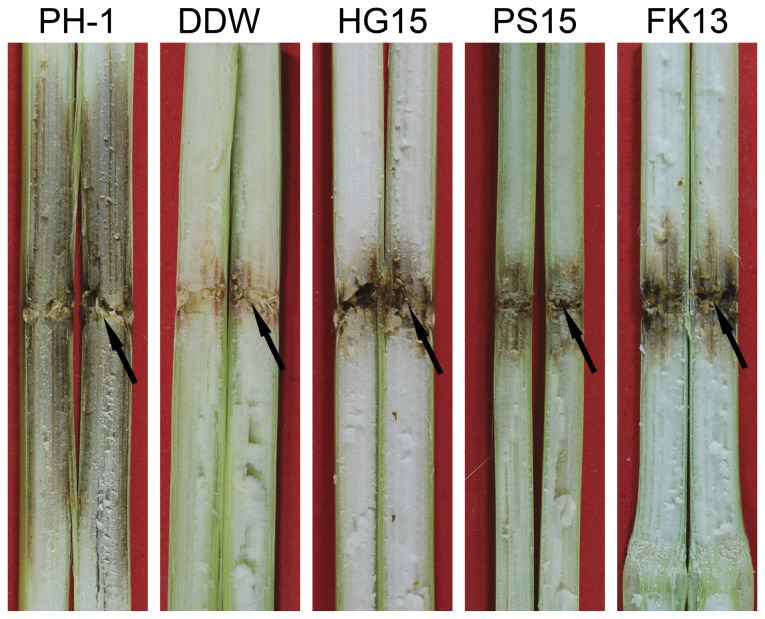
Corn stalks inoculated with the wild type (PH-1) and the *Fghog1* (HG15), *Fgpbs2* (PS15), and *Fgssk2* (FK13) mutants were examined 10 dpi. Arrows pointed to the inoculation sites.

On wheat heads, the disease index was 0.5, 0.8, 1.5, respectively for the *Fgssk2*, *Fgpbs2*, and *Fghog1* mutant. Normally, only the inoculated wheat kernels developed symptoms. In contrast, the *Fghog1*/*FgHOG1* complemented transformant was normal in virulence. Microscopic examination revealed that the *Fghog1* mutant still colonize the glume tissues (Fig. 5A) but failed to observe fungal growth in the rachis (Fig. 5B). These results indicate that the FgHog1 pathway mutants could penetrate and colonize plant tissues but were defective in spreading through tissues in wheat heads after the initial infection. In corn infection assays, mutants blocked in the FgHog1 MAPK pathway also were significantly reduced in virulence but still caused stalk rot at the wounding sites (Fig. 6). These data further indicate that the *FgHOG1* pathway is important for plant infection. Because DON is one of the best characterized virulence factors in *F. graminearum* [22], we assayed DON production in the *Fgssk2*, *Fgpbs2*, and *Fghog1* mutants. In the rice grain cultures, all the FgHog1 MAPK pathway mutants were reduced in DON production (Table 2).

### Expression and localization of FgHog1-GFP

**Figure 7:**
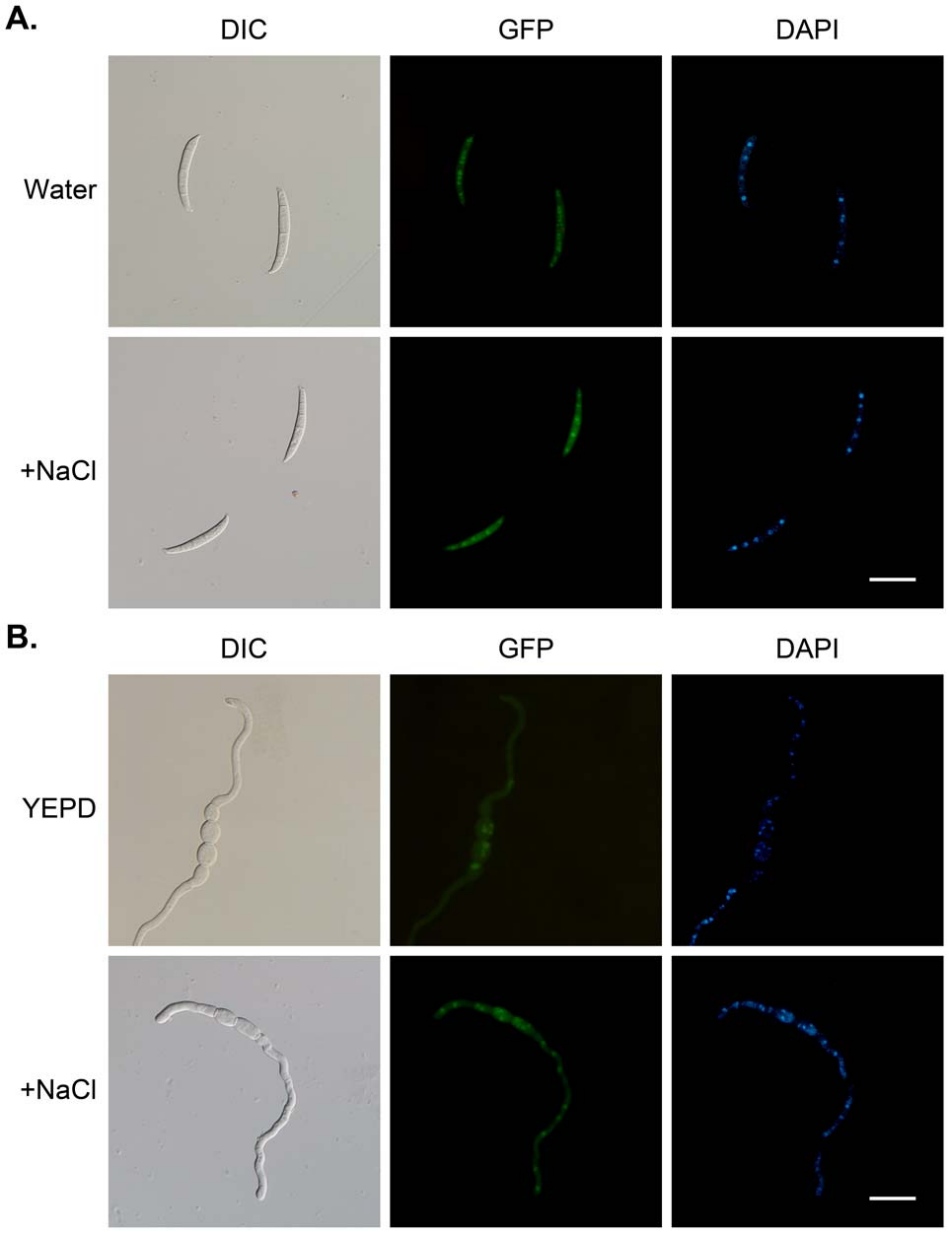
Expression and subcellular localization of FgHog1-GFP. **A.** Conidia harvested from the *Fghog1*/*FgHOG1*-GFP transformant HGC1 were re-suspended in sterile distilled water or 0.3 M NaCl and examined by DIC or epifluorescence microscopy (GFP). **B.** GFP signals in germlings of FGC1 were incubated in the liquid YEPD medium with or without 0.3M NaCl. Nuclei were stained with DAPI. Bar = 20 µm.

To confirm the mutant phenotypes, an *FgHOG1*-GFP construct was generated and transformed into the *Fghog1* mutant. The resulting *Fghog1/FgHOG1*-GFP transformant was normal in hyphal growth, conidiation (Table 2), and plant infection (Fig. 5A), indicating that *FgHOG1*-GFP complemented the defects of the *Fghog1* mutant. In conidia of the *FgHOG1*-GFP transformant, GFP signals were mainly observed in the cytoplasm. In the presence of 0.3M NaCl, however, GFP signals were enhanced in the nucleus (Fig. 7A). In contrast, germlings of the *FgHOG1*-GFP transformant had detectable GFP signals in the nucleus without NaCl treatment (Fig. 7B). Nevertheless, GFP signals in the nucleus were stronger when the *FgHOG1*-GFP transformant was under hyperosmotic stress (Fig. 7B), suggesting that FgHog1 was activated and localized to the nucleus by NaCl treatment.

### Deletion of *FgSSK2* or *FgPBS2* blocked the activation of FgHog1 but not Gpmk1 or Mgv1

**Figure 8:**
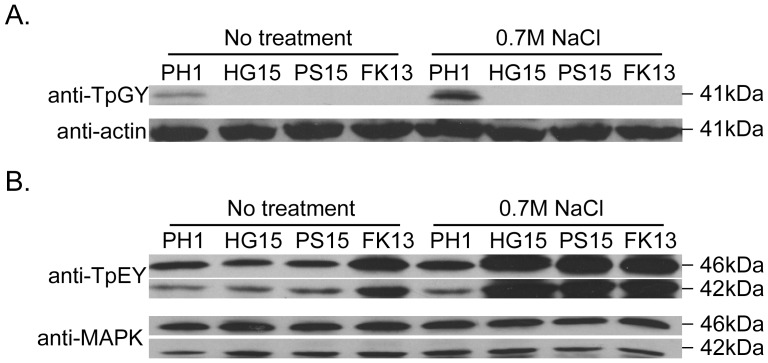
Assays for the activation of FgHog1, Mgv1, and Gpmk1 MAP kinases. Total proteins were isolated from vegetative hyphae of the wild type (PH-1) and the *Fghog1* (HG15), *Fgpbs2* (PS15), and *Fgssk2* (FK13) mutants. **A.** The anti-TpGY antibody was used to detect the phosphorylation of FgHog1 (41-kDa) in cultures treated with or without 0.7 M NaCl. **B.** Phosphorylation of Mgv1 (46-kDa) and Gpmk1 (42-kDa) was detected with the anti-TpEY antibody. Anti-actin and anti-MAPK antibodies were used to determine the same loading amount of protein.

Based on their orthologs in the budding yeast and mutant phenotypes, *FgSSK2* and *FgPBS2* likely function upstream *FgHOG1* in *F. graminearum*. To experimentally confirm this hypothesis, we isolated proteins from vegetative hyphae grown in YEPD and assayed for the phosphorylation of FgHog1. When assayed with the anti-TpGY antibody, a 41-kDa band was detected only in proteins isolated from PH-1 (Fig. 8A). Like the *Fghog1* deletion mutant, the phosphorylation of FgHog1 was not detectable in the *Fgssk2* and *Fgpbs2* mutants (Fig. 8A). Therefore, FgSsk2 and FgPbs2 must function upstream from the FgHog1 MAP kinase.

We also assayed the activation of Mgv1 and Gpmk1, the two other MAPKs in *F. graminearum*, in the FgHog1 MAPK pathway mutants. When cultured under normal conditions, the phosphorylation level of Gpmk1 or Mgv1 detected with the anti-TpEY antibody was similar between the wild type and *Fghog1* and *Fgpbs2* mutants. However, the *Fgssk2* mutant appeared to have enhanced activation of Gpmk1 and Mgv1. In the presence of 0.7 M NaCl, the activation level of Mgv1 and Gpmk1 was significantly increased in all three FgHog1 MAPK pathway mutants than in the wild type (Fig. 8B).

### The accumulation of glycerol, arabitol, mannitol, and sucrose was not induced by NaCl treatment in the *Fghog1* mutant

**Figure 9:**
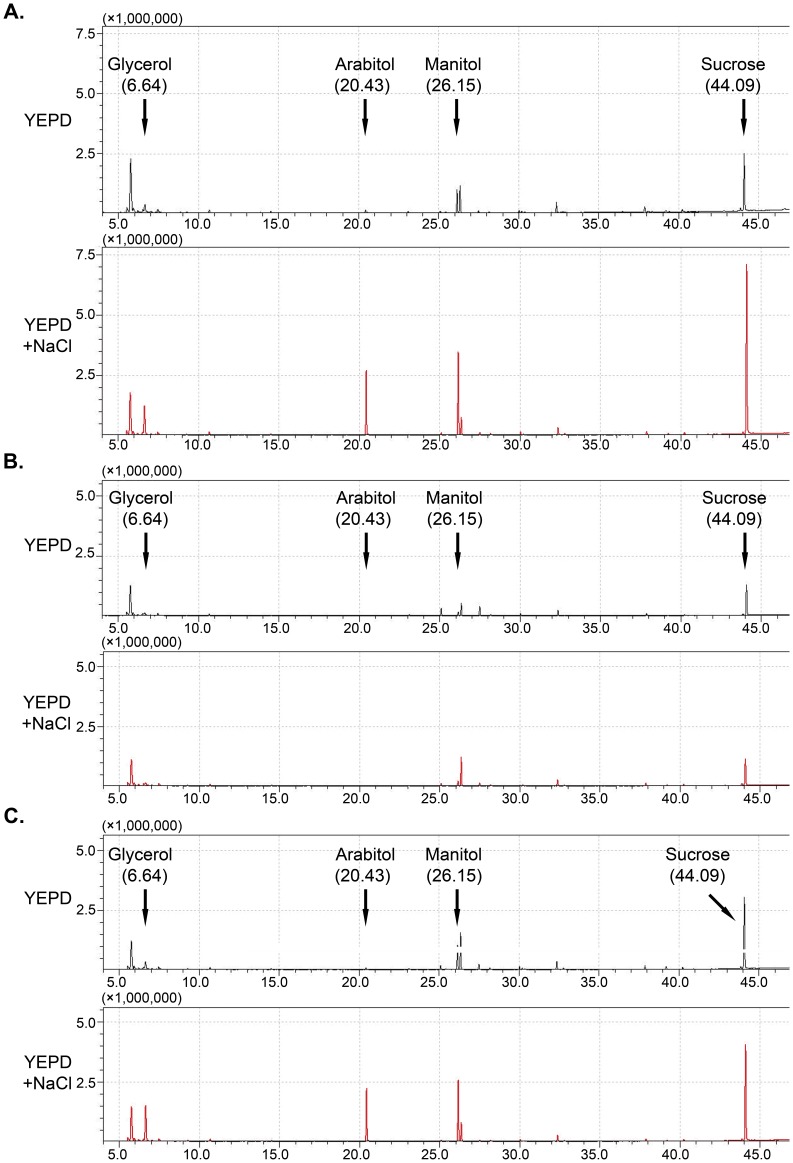
Metabolic profiles of the wild type PH-1 (**A**), *Fghog1* mutant (**B**), and the *Fghog1*/*FgHOG1* complemented transformant (**C**) cultured in YEPD with or without 1 M NaCl. The X-axis is the retention time (RT) in minutes. The Y-axis represents the abundance of total ion current. The peaks with RT of 6.64, 20.43, 26.15, and 44.09 are glycerol, arabitol, mannitol, and sucrose, respectively. The far left peak (RT = 5.75) is 1,2,2,3,4,5-hexamethyl-1,2,5-azasilaborole.

Several neutral compatible solutes have been reported to be accumulated in different fungi in response to hyperosmotic stress [23]. To determine which compatible solutes were accumulated in *F. graminearum* in response to hyperosmotic stress, we compared metabolites in cultures of the wild-type strain PH-1 treated with or without 1 M NaCl. Four metabolites with retention time (RT) of 6.64 (glycerol), 20.43 (arabitol), 26.15 (mannitol), and 44.09 (sucrose) were induced by NaCl treatment in the wild type (Fig. 9A). In the *Fghog1* mutant, the production of glycerol and arabitol was not or barely detectable in hyphae harvested from YEPD or YEPD with 1 M NaCl (Fig. 9B). However, mannitol and sucrose were still accumulated in the mutant but to a much lower level than that of the wild type, even in the presence of 1 M NaCl (Fig. 9B), suggesting that hyperosmotic stress failed to induce their accumulation in the *Fghog1* mutant (Fig. 9B). In the complemented transformant HGC1, the production of these four compounds with or without NaCl treatment was similar to that of the wild type (Fig. 9C). These results indicate that the accumulation of glycerol, arabitol, mannitol, and possibly sucrose as compatible solutes in response to hyperosmotic stress is under the control of the FgHog1 MAP kinase pathway in *F. graminearum*.

## Discussion

In yeast and filamentous fungi, the Hog1 MAPK pathway has been shown to be well conserved for regulating responses to hyperosmotic stresses [24]. It is not surprising that the *Fgssk2* ( = *FgOs4*), *Fgpbs2* ( = *FgOs5*), and *Fghog1* ( = *FgOs2*) mutants were hypersensitive to osmotic stress in *F. graminearum* as previously reported [19]. However, to our knowledge, the involvement of this MAPK pathway in the regulation of hyphal growth/branching under normal culture conditions has not been reported in filamentous fungi. In addition to reduced growth rate, aerial hyphae and surface hydrophobicity were reduced in the mutants blocked in the FgHog1 pathway. Vegetative hyphae of these mutants also had smaller branching angles and tended to grow in parallel in the extension zone of colonies formed on PDA plates, suggesting that the FgHog1 pathway may be involved in the negative autotropism of hyphal growth under certain growth conditions in *F. graminearum*.

Interestingly, colonies formed by the FgHog1 MAPK pathway mutants had distinct morphology on PDA plates (Fig. 1). Because adding components of 5xYEG to PDA had no obvious effects, we concluded that the distinct growth and colony morphology defects of the FgHog1 pathway mutants on PDA were not related to nutritional conditions. During the revision of this manuscript, the *Fgos2* ( = *Fghog1*) deletion mutant was reported to be sensitive to lower pH [25]. However, the FgHog1 pathway mutants displayed the same growth defects on PDA buffered at pH 6.8 with 0.1 M PBS. As a natural medium,

In *F. graminearum*, other protein kinase genes that may be involved in response to 0.7 M NaCl include FGSG_04382, FGSG_06939, FGSG_09274 that are orthologous to yeast *FPK1*, *SAT4*, and *KIN1*, respectively [20]. Like the FgHog1 MAPK pathway mutants, the *FgFPK1* deletion mutant had no detectable hyphal growth on media with 0.7 M NaCl or 1 M sorbitol [20]. Therefore, proper regulation of phospholipid translocation by FgFpk1 kinase also plays a role in normal response to hyperosmotic stress in *F. graminearum*. On the other hand, deletion of the *FgSAT4* and *FgKIN1* genes resulted in increased sensitivity to 0.7 M NaCl but not 1 M sorbitol. Furthermore, the *Fgsat4* deletion mutant was more tolerant to 0.7 M KCl than the wild type [20]. Therefore, increased sensitivity to 0.7 M NaCl in the *Fgsat4* and *Fgkin1* mutants must be caused by their defects in functions different from the osmoregulation pathway. Orthologs of these two protein kinase genes have not been characterized in filamentous fungi but the Sat4 kinase regulates potassium transporters in *S. cerevisiae*.

In several fungi, including *C. lagenarium* and *B. cinerea*, the osmoregulation pathway also regulates responses to oxidative stress [14], [26]. In *F. graminearum*, the *Fgssk2*, *Fgpbs2*, and *Fghog1* mutants were slightly more sensitive to H_2_O_2_ than the wild type. However, recently the *Fgos2* mutant was reported to be more resistant to H_2_O_2_ [25]. The difference between the *Fgos2* mutant [25] and the FgHog1 pathway mutants characterized in this study in response to oxidative stress may be caused by different strain background or experimental conditions. In addition to H_2_O_2_, we found that the *Fgssk2*, *Fgpbs2*, and *Fghog1* mutants also were more sensitive to tert-butyl hydroperoxide than PH-1 (Fig. S2). These mutants also had increased sensitivity to SDS and Congo red (Fig. 3), indicating that the FgHog1 pathway may be involved in general stress responses in *F. graminearum*. Nevertheless, NaCl treatment increased the activation of Mgv1 and Gpmk1 in the *Fghog1* mutant (Fig. 8). Some of the observed changes in stress responses may be related to cross-talking among these MAPK pathways, which has been reported in several filamentous fungi, particularly between the osmoregulation and cell wall integrity pathways [15], [27], [28], [29].

The osmoregulation pathway varies significantly among different fungal pathogens for its function in plant infection. Whereas it is dispensable in *M. oryzae*, the HOG pathway is essential for pathogenicity in several other fungi, including *M. graminicola* [12], *B. cinerea* [13] and *Cryphonectria parasitica*, [30]. We found that the disease index of the FgHog1 MAPK pathway mutants was less than 1.5 and the *Fghog1* mutant was defective in spreading along the wheat rachis (Fig. 5), indicating that FgHog1 plays a critical role in pathogenesis, which was not reported in the earlier study [19]. During the revision of this manuscript, the *FgOS2* gene was shown to be important for plant infection [25]. The *Fgssk2*, *Fgpbs2*, and *Fghog1* mutants were slightly reduced growth rate and increased in sensitivity to cell wall stresses. In addition, these FgHog1 MAPK pathway mutants had increased sensitivities to H_2_O_2_. Oxidative burst is a common plant defense response. Therefore, it is likely that defects in general stress response may contribute to reduced virulence in the *Fghog1* mutant. However, DON production is an important virulence factor in *F. graminearum*. In rice grain cultures, these mutants were reduced in DON production, which was consistent with previous reports with the *FgOS2* and *FgRRG1* deletion mutants [19], [31]. Similar to the Mgv1 and Gpmk1 MAPKs [32], [33], [34], FgHog1 may be somehow indirectly involved in the regulation of DON biosynthesis in *F. graminearum*.

In *S. cerevisiae*, the Pbs2-Hog1 MAPK module is mainly activated by Ssk2 and Ssk22, which are two MEKKs with overlapping functions. *F. graminearum*, like other filamentous fungi, has only one ortholog of Ssk2 and Ssk22. In this study, we found that the *Fgssk2* and *Fgpbs2* mutants had similar phenotypes with the *Fghog1* mutant. In the *Fgssk2* and *Fgpbs2* mutants, the activation of FgHog1 was blocked. These results indicate that FgSsk2 and FgPbs2 function upstream from FgHog1. In yeast, the Sho1-Ste11 branch also plays a role in the activation of downstream Pbs2-Hog1 [35]. The genome of *F. graminearum* contains one putative ortholog of Sho1 (FGSG_09435), which has not been functional characterized. Nevertheless, although they had the same defects in hyperosmoregulation, pathogenesis, and sexual reproduction, the *Fgssk2* mutant had some minor phenotype difference in colony morphology and surface hydrophobicity with the *Fgpbs2* and *Fghog1* mutants. Therefore, the Sho1 branch likely also functions upstream from FgPbs2-FgHog1 in response to certain but not all stresses.

Glycerol production and accumulation is well known to be regulated by the HOG pathway in *S. cerevisiae*. Our data indicated that glycerol production also is regulated by FgHog1 in *F. graminearum*. We also found that the accumulation of arabitol, mannitol, and sucrose was induced by NaCl treatment in the wild type but not in the *Fghog1* mutant (Fig. 9). Whereas the production of arabitol was not detectable, mannitol and sucrose were still accumulated in mutant cultures grown under normal conditions. However, NaCl treatment failed to induce the production of these compounds in the *Fghog1* mutant, indicating that arabitol, mannitol, and sucrose also are accumulated as compatible solutes in *F. graminearum* in response to hyperosmotic stress. In summary, our data indicate that the FgHog1 MAPK pathway regulates hyphal growth and branching in *F. graminearum*. It also plays a critical role in plant infection and sexual reproduction. Therefore, it will be important to further characterize the functions and downstream targets of this MAPK pathway in hyphal growth, sexual reproduction, and pathogenesis.

## Materials and Methods

### Strains and culture conditions

The wild-type strain PH-1 and mutants of *F. graminearum* were routinely cultured on complete medium (CM), potato dextrose agar (PDA, Sigma, St. Louis, MO), or 5xYEG plates at 25°C as described [34], [36]. The *MAT1-2* deletion mutant FgMAT2 was generated with the split marker approach with primers 1F (TGTACGCATCCCG CCTCAAGAT), 2R (TTGACCTCCACTAGCTCCAGCCAAGCCCCTGCCCCACACACAA TTCAG), 3F (GAATAGAGTAGATGCCGACCGCGGGTTACAAGGACAACGGGCGGTA ATGT), and 4R (ACCCCACCACCGAGATTGATCTG). For testing sensitivities to various stresses, fungal growth was assayed after incubation at 25°C for four days on CM plates with 1 M sorbitol, 0.7% NaCl (w/v), 0.01% SDS (w/v), 0.05% H_2_O_2_, 300 mM Congo Red or 1 mM tert-butyl hydroperoxide (Alfa Aesar). Conidiation was assayed with 5-day-old CMC (15 g carboxymethyl cellulose, 1 g Yeast Extract, 0.5 g MgSO_4_, 1 g NH_4_NO_3_, and 1 g KH_2_PO_4_) cultures as described [37], [38], [39]. Freshly harvested conidia were resuspended to 5×10^5^ conidia/ml in CM with or without 0.7 M NaCl, 1 M KCl, or 1 M sorbitol and assayed for germination after incubation for 6, 12, and 18 h. Surface hydrophobicity was assayed by adding droplets of 20 µl of red ink solution onto 3-day-old CM cultures as described [40]. Protoplast preparation and PEG (polyethylene glycerol)-mediated transformation of *F. graminearum* were performed as described [22], [34]. For selection of transformants, hygromycin and geneticin (Sigma) were added to the final concentration of 250 and 150 µg/ml, respectively, to both bottom and top agars. Autoclaved rice grains were inoculated with conidia of the wild type and mutant strains, cultured at room temperature for 10 days, and assayed for DON and ergosterol as described [41], [42].

### Assays for defects in sexual reproduction

For self-crossing, aerial hyphae of 7-day-old carrot agar cultures of the *Fgssk2*, *Fgpbs2*, and *Fghog1* mutants were pressed down with 300 µl of sterile 0.1% Tween 20 as described [43]. For outcrossing, 7-day-old carrot agar cultures of the *mat2* deletion mutant were fertilized with conidium suspensions (1×10^7^ conidia/ml) of the wild-type and mutant strains. Fertilized plates were cultured at 25°C under 12/12 h light and dark cycle. Perithecium formation and cirrhi production were examined 1week after fertilization.

### Plant infection assays

Conidia harvested from 5-day-old CMC cultures were re-suspended to 10^5^ spores/ml in sterile distilled water. Wheat heads of cultivars Norm or Xiaoyan22 were inoculated with 10 µl of conidium suspensions at the fifth spikelet from the base of the spike as described [44]. After the inoculation, wheat heads were capped with a plastic bag for 48 h to maintain the moisture. Spikelets with typical symptoms were examined 14 days post-inoculation (dpi). Infested wheat kernels were collected and assayed for DON production as described [36], [42]. For microscopic examinations, inoculated spikeletes were collected and embedded in Spurr resins [45]. Thick sections (1 µm) were stained with aqueous 0.5% (w/v) toluidine blue before examination. For corn stalk rot assays, stalks of 8-week-old plants of cultivar Pioneer 2375 were punctured with toothpicks dipped in conidium suspensions as described [37], [46]. Symptom development was observed after splitting the stalks along the inoculation sites 14 dpi.

### Generation of the *FgHOG1*-GFP fusion construct and transformant

For complementation assays, a 3.1-kb fragment of the *FgHOG1* gene (containing the 1.5-kb promoter region) was amplified and cloned into pFL2 [47] by the yeast in vivo homologous recombination approach as described [48]. The resulting *FgHOG1*-GFP construct pDW2 was transformed into the *Fghog1* deletion mutant HG15. Transformants expressing the *FgHOG1*-GFP construct were analyzed by PCR to contain the transforming *FgHOG1*-GFP fusion. GFP signals in conidia and germlings were observed with an Olympus BX51 epifluorescence microscope (Olympus Corporation, Japan). Nuclei were stained with DAPI as described [49].

### Assays for the expression and phosphorylation of MAP kinases by western blot analysis

Hyphae harvested from two-day-old YEPD cultures of the wild-type and mutant strains were further incubated in YEPD with or without 0.7 M NaCl for 30 minutes. Total proteins were then isolated from hyphae, separated on 12.5% SDS-PAGE gels, and transferred to nitrocellulose membranes [48], [50]. TEY-phosphorylation of Mgv1 or Gpmk1 were detected with the PhophoPlus p44/42 antibody kit (Cell Signaling Technology, Danvers, MA) as described [51]. The PhophoPlus p38 MAP kinase antibody kit (Cell Signaling Technology) was used to detect TGY- phosphorylation of FgHog1 following the manufacturer's instructions.

### Analysis for compatible solutes

Hyphae harvested from 200 ml of two-day-old YEPD cultures were split into halves. One halve was incubated with regular YEPD and the other half incubated in YEPD with 1 M NaCl. After incubation at 25°C for 30 min, hyphae were harvested by filtering through Miracloth and rinsed with sterile distilled water. The samples were then ground in liquid nitrogen and dried for 24 h in a freeze dryer. Six milligrams of ground hyphal tissues were transferred into a 4 ml autosampler vial, suspended in 2 ml methanol, and incubated overnight at room temperature. After centrifugation at 4,000 rpm for 10 min, 100 µl of the supernatant was transferred to a conical vial and dried under a gentle nitrogen stream. The content was then re-suspended in 100 µl of 1 M HCl, incubated at 50°C for 1 h, dried under a nitrogen stream as described [52], [53]. The solid extract was re-suspended in 100 µl of TMSI∶TMCS (100∶1), incubated at 37°C for 1 h, then mixed with 300 µl of isooctane and 300 µl Type I water. After the aqueous and organic layers were completely separated, and the top (organic) phase was analyzed with a GCMS-QP2010 (Shimadzu, Japan) in the scan mode (scanning from m/z 40 to 650). Helium was used as the carrier gas at a constant flow rate of 1 mL/min through an Rxi-5ms (30 m×0.25 mm, 0.25 µm) capillary column (Restrek, Bellefonte, USA) with the injector temperature of 260°C and split ratio of 1∶5.

## Supporting Information

Fig. S1 **Growth and colony morphology defects on PDA+5xYEG plates.** Nutritional components of 5xYEG were added to PDA to the final concentrations of regular 5xYEG medium. Colonies formed by the wild type (PH-1), *Fghog1* (HG15), *Fgpbs2* (PS15), and *Fgssk2* (FK13) mutants, and complemented transformant (HGC1) were photographed after incubation for 3 days.
(TIF)

Fig. S2 **Growth and colony morphology defects of the** ***Fghog1*** **,** ***Fgpbs2*** **, and** ***Fgssk2*** **mutants on PDA plates with 1 mM tert-butyl hydroperoxide (TBOOH).** Photographs were taken after incubation at 25°C for 4 days.
(TIF)
