# Supplementary figures and images for: The FgHOG1 Pathway Regulates Hyphal Growth, Stress Responses, and Plant Infection in Fusarium graminearum
Source: PLoS One. 2012 Nov 14;7(11):e49495. doi: 10.1371/journal.pone.0049495 (PMC3498113; doi:10.1371/journal.pone.0049495)

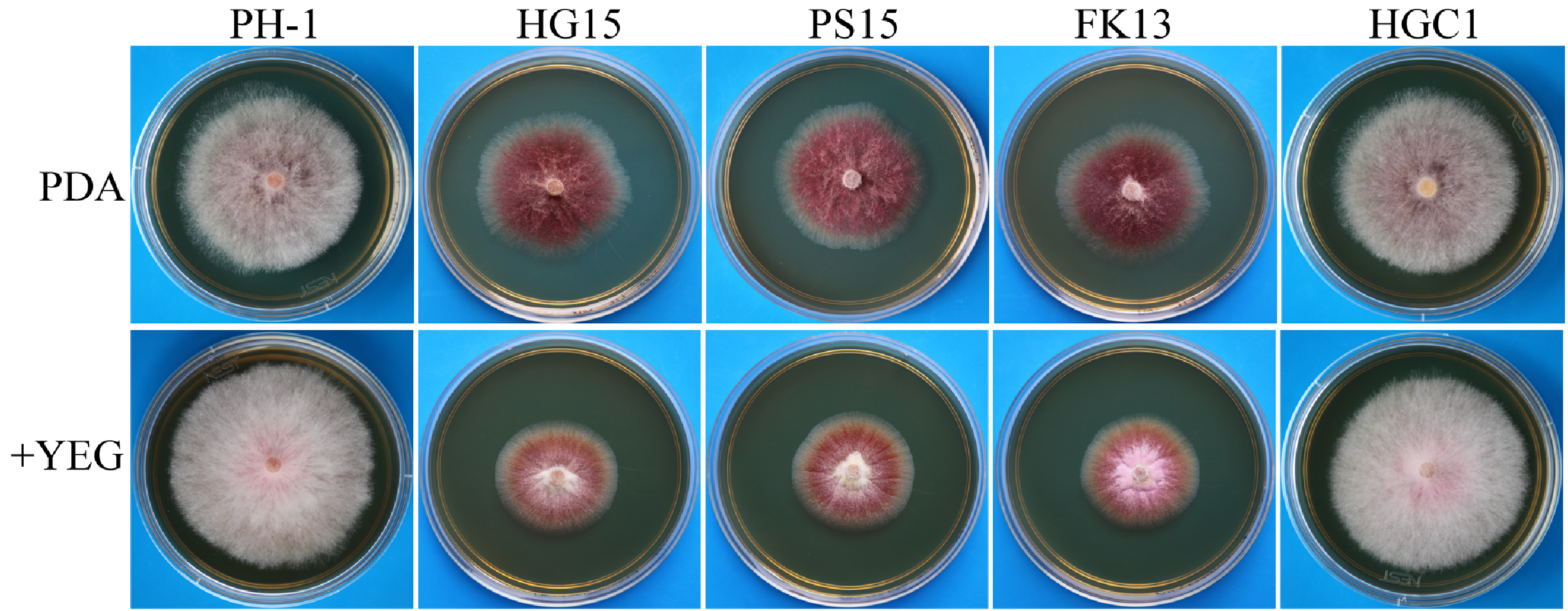

Supplement: Fig. S1 — Growth and colony morphology defects on PDA+5xYEG plates. Nutritional components of 5xYEG were added to PDA to the final concentrations of regular 5xYEG medium. Colonies formed by the wild type (PH-1), Fghog1 (HG15), Fgpbs2 (PS15), and Fgssk2 (FK13) mutants, and complemented transformant (HGC1) were photographed after incubation for 3 days. (TIF) [file pone.0049495.s001.tif]

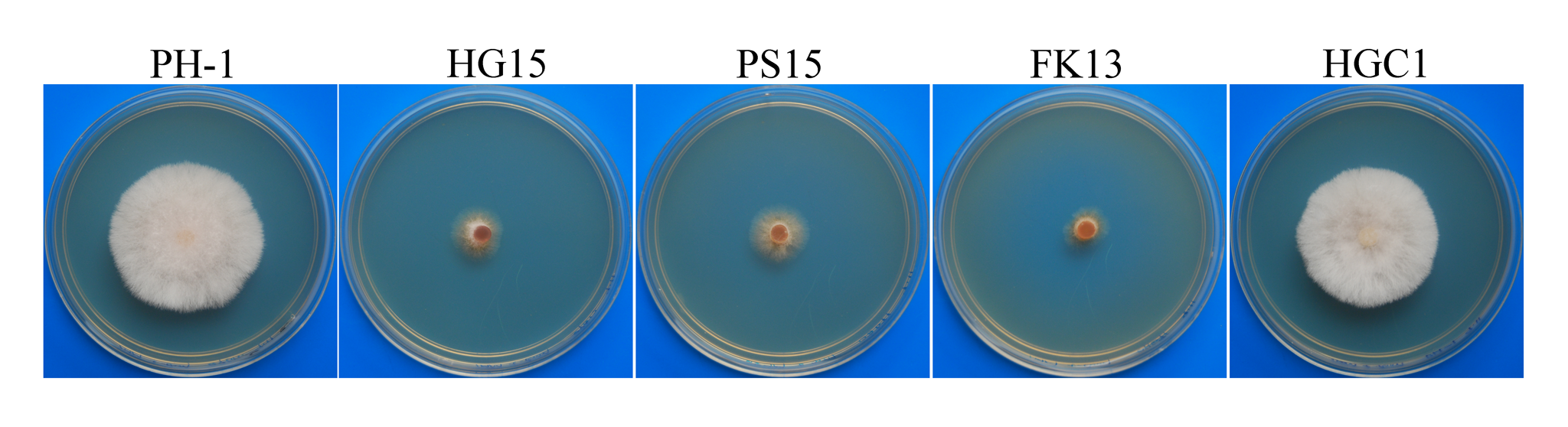

Supplement: Fig. S2 — Growth and colony morphology defects of the Fghog1 , Fgpbs2 , and Fgssk2 mutants on PDA plates with 1 mM tert-butyl hydroperoxide (TBOOH). Photographs were taken after incubation at 25°C for 4 days. (TIF) [file pone.0049495.s002.tif]
